# Mother’s nutrition-related knowledge and child nutrition outcomes: Empirical evidence from Nigeria

**DOI:** 10.1371/journal.pone.0212775

**Published:** 2019-02-28

**Authors:** Olusegun Fadare, Mulubrhan Amare, George Mavrotas, Dare Akerele, Adebayo Ogunniyi

**Affiliations:** 1 International Food Policy Research Institute, Development Strategy and Governance Division, Washington, DC, United States of America; 2 Federal University of Agriculture, Abeokuta, Nigeria; BITS Pilani, INDIA

## Abstract

**Background:**

Nutrition outcomes among young children in Nigeria are among the worse globally. Mother’s limited knowledge about food choices, feeding, and health care seeking practices contributes significantly to negative nutrition outcomes for children in most developing countries. Much less is known about the relationship between mother’s nutrition-related knowledge and child nutritional outcomes in rural Nigeria. This paper investigates therefore: (i) the association of mother’s nutrition-related knowledge with nutrition outcomes of young children living in rural Nigeria, where access to education is limited, and (ii) whether mother’s education has a complementary effect on such knowledge in producing positive child nutrition outcomes in such settings.

**Methods:**

Using the Demographic and Health Survey data for Nigeria, we employ both descriptive and regression analyses approaches in analyzing the study’s objectives. In particular, we apply ordinary least square (OLS) to investigate the association of mother’s nutrition-related knowledge with child HAZ and WHZ while controlling for maternal, child, household and regional characteristics. An index was constructed for mother’s nutrition-related knowledge using information on dietary practices, disease treatment and prevention, child immunization, and family planning.

**Results:**

We found that mother’s knowledge is independently and positively associated with HAZ and WHZ scores in young children. Higher levels of mother’s education, typically above primary, have a significant, positive association with child HAZ and WHZ scores. We argue that mother’s knowledge of health and nutrition may substitute for education in reducing undernutrition in young children among populations with limited access to formal education. However, the present level of mother’s education in rural Nigeria appears insufficient to reinforce knowledge in producing better nutrition outcomes for children.

**Conclusions:**

This study suggests promotion of out-of-school (informal) education, such as adult literacy and numeracy classes where women without formal education can gain health and nutrition knowledge, and practices that could enhance child nutrition outcomes in Nigeria.

## Introduction

Child nutrition outcomes, stunting and wasting in particular, are recognized as key indicators for tracking the nutrition and health status of children in a population [1]. More countries now recognize the need to give priority to policies and programs that improve mothers’ ability to provide optimal care for young children, especially during the period from pregnancy to a child’s second birthday [2]. This period presents a critical window of opportunity for parents to use both human and material resources for child growth and cognitive development [3–5]. Empirical evidence, however, suggests that availability of resources such as food and health facilities, and their accessibility, including location advantage, are not sufficient to produce child health [6–8].

More consideration is being given to human resource determinants, such as mother’s education, and in particular mother’s knowledge in relation to food choices, feeding and health seeking [9–15]. Against this background, the present study investigates: (i) whether mother’s nutrition-related knowledge (mother’s knowledge) can substitute for mother’s education or is complementary in producing positive child nutrition outcomes in a population where access to education is limited (for example, rural Nigeria); and (ii) the education level at which mother’s education is positively associated with child nutrition outcomes. To the best of authors’ knowledge, only a limited number of studies on Nigeria have made an attempt to empirically investigate the objectives of this study. More importantly, the use of a nationally representative data, the Demographic and Health Survey (DHS) for Nigeria [16], positions this study to be useful in the design of policies for less educated population and girl-child education in Nigeria or elsewhere with similar characteristics. We hypothesize that in rural Nigeria mother’s nutrition-related knowledge contributes independently to height-for-age z-scores (HAZ) and weight-for-height z-scores (WHZ), and that mother’s education is associated with these nutritional outcome indicators at a level above primary school.

Nutritional outcomes among under-five children in Nigeria are among the worse globally [2]. More than one third of children under five years of age in Nigeria are stunted (i.e. too short for their age or with height for age <-2), which represents about 9.7 million stunted Nigerian children in 2013 [16, 17]. Likewise, approximately 18% of under-five children are wasted (i.e. too thin for their height or with weight for height <-2) in the same year. The prevalence of child undernutrition is higher for less educated mothers, with about half of children of less educated mothers reported stunted in 2013. In addition, school attendance rate and educational attainment for women are very low in rural Nigeria. More than half of women in rural Nigeria never attended school, and higher percentage of girls drop out of school before they attain 15 years of age [16], thereby denying them the basic knowledge of health and nutrition [18]. In Nigeria, primary education does not include element of public health nutrition, but course on nutrition is part of curricula in the junior secondary [18]. This work will contribute to the limited in-depth studies on education of mothers as a key driver of child malnutrition in Nigeria.

The objectives presented in this study present a relationship that most research on developing countries have not explained empirically [19–22]. Specifically, the paper explores the association of mother’s nutrition-related knowledge with nutritional outcomes of young children living in rural Nigeria, where access to education is limited. Our explanatory variables are based on a strong conceptual understanding of the determinants of child undernutrition in literature. Our model examines the determinants of HAZ and WHZ among young children (6–23 months).

Research in this area continues to delve deeper into the complex relationship between mother’s knowledge of nutrition and/or health and mother’s education in producing child health. Most studies have found a strong association between education and child nutrition outcomes after controlling for other confounders [23, 24]. Other studies indicate that the effect of mother’s education on child health (infant mortality and stunting) is attenuated or lost significance once major confounders such as socioeconomic and geographic characteristics are controlled for [25–29]. More studies [30, 31] show no significant relationship between parental education (lower education level) and child nutritional outcomes. The effect tends to evolve when parents have beyond primary level of education. The studies suggest that community-based education on maternal nutrition knowledge for young and out-of-school women could substitute for low level of parental education in producing children with better nutrition outcomes in developing counties at least for short run [32, 33].

Furthermore, studies on the double burden of malnutrition–a condition where undernutrition and over-nutrition coexist in a population [34, 35] in low-income countries–suggest that malnutrition is more than just a lack of physical or economic access to food. Limited knowledge of appropriate food choices and feeding and healthcare practices also play a huge role. In a multi-country meta-analysis of the impact of cash transfers on child nutrition status (height-for-age), [36, 37] studies found no statistically significant association between cash transfers and stunting reduction. This suggests that in the absence of good knowledge of childcare, resources may not be optimally utilized to benefit children’s health.

## Materials and methods

### Data

The 2013 DHS data employed for this study is the most recent one for Nigeria. The sample was selected using a stratified three-stage cluster design consisting of 904 clusters, with a total of 38,522 households. Sampling errors are computed statistically using appropriate tools and methodologies as provided in the material and methodology document of the 2013 Nigeria Demographic and Health Survey [16]. The children module of the DHS comprises of 31,482 children under five years with their mothers between 15 to 49 years. Children living in rural households are 21,131 (67%), and 5,950 of them are between 6 to 23 years. We only included the youngest child between 6 to 23 months per family, and following deletion of observations with incomplete anthropometric measures, 4,941 mother-child pairs were finally available for analysis. The dataset captures mothers, children, and household information such as socioeconomic characteristics, anthropometric measurements for mother and child, immunization records, health care seeking and feeding practices, water and sanitation, and demographic information among others.

Children are being introduced to complementary feeding between 6–23 months, as such, they are the most vulnerable group to undernutrition and consequent growth faltering [3, 37]. Hence, children in this age bracket benefit the most when their mothers have basic knowledge of health and nutritional care. This is a very important, as the consequences of undernutrition, stunting in particular are difficult to reverse in children after the age of two.

### Young child nutrition outcomes

Height-for-age z-scores (HAZ) and weight-for-height z-score (WHZ) are good indicators of child nutrition and health. Children with HAZ less than two standard deviations below the median measurement for the reference group are said to be stunted or chronically undernourished. While children with WHZ less than two standard deviations below the median measurement for the reference group are regarded as wasted or acutely undernourished [38]. Hence, these two indicators measure whether a child is undernourished (stunted or wasted) or not. It is of policy relevance to investigate separately the factors producing wasting and stunting gauging from these indicators.

### Determinants of young child nutrition outcomes (HAZ and WHZ)

Our choice of the determinant variables was informed by the literature on the determinants of child nutrition outcomes [39]. We included child and mother characteristics and household characteristics. Child characteristics are sex, age, birthweight and child from multiple birth. Mother characteristics include age at first birth, number of children ever born, mother’s level of education and nutrition and health knowledge of mother (an index computed). Household characteristics are household size; education; age; and an asset-based wealth index, among others. An asset-based wealth index is constructed using a principal components analysis of the NDHS assets data, combining variables on ownership of a radio, bicycle, car, and other items with household dwelling characteristics [40]. This allows households to be ranked by wealth index. We ranked households as poorest tercile wealth index, middle tercile wealth index and wealthiest tercile wealth index. The region dummies where the household belongs, which comprises of the six geopolitical regions of Nigeria. Under this sub-heading, we discuss the computation of mother’s nutrition-related knowledge index, the measurement of mother’s education variable and its interaction with mother’s knowledge.

### Mother’s nutrition-related knowledge index

The DHS data contains some important information on dietary practices, disease treatment and prevention, child immunization and family planning. We follow the guidelines for assessing nutrition-related knowledge, attitudes, and practices (KAP) as contained in [41]. Mother’s nutrition-related knowledge was assessed based on five key nutrition and health information as follows: (i) mother’s knowledge of the important of colostrum; (ii) knowledge of continued breastfeeding; (iii) knowledge of diarrhea prevention and treatment using Oral Rehydration Solution (ORS); (iv) knowledge of child immunization; and (v) knowledge of family planning. A detailed description of these variables and their measurement is presented in the result section.

Past studies have assessed mother’s nutrition knowledge by either assigning scores to observed knowledge (practice) [42] or scoring mother’s ability to answer “yes” or “no” to a set of questions relating to child health and nutrition [43, 44] or a combination of these [33]. A strong linear relationship between knowledge of young child nutrition and practices has been established in the literature, especially where there are no sociocultural barriers to such practices [45, 46]. We then apply principal component analysis (PCA) as adapted from Filmer and Pritchett [47] to construct the mother’s Nutrition Knowledge Index using the five components highlighted above. Variables for each component are assigned indicator weights that are first standardized; that is, z-scores are calculated and then factor coefficient (factor loading) scores are calculated. More details are shown in S1 File.

### Mother’s education

Since this study is interested in knowing at what level of education is the association of mother’s education most significant with nutrition outcomes, mother’s levels of educational attainment are categorized as follows in the empirical model used: no education, primary education, secondary education, and tertiary education.

### Mother’s nutrition-related knowledge versus mother’s education variables

We further used the interaction between the four educational level dummy variables and mother’s knowledge index to produce four interaction terms. Adding interaction terms to the model helps to better understand the relationship between knowledge and education and the association with outcomes. In other words, to test whether the association of mother’s knowledge with HAZ and WHZ is different at the different levels of mother’s education. For the purpose of nonparametric analysis between mother’s knowledge versus mother’s education on the distribution of HAZ and WHZ scores, we defined mothers with high knowledge as those with above the mean of nutrition-related knowledge in our sample, while mothers with low knowledge are below the mean cut off for nutrition-related knowledge index. For the purpose of interaction model, we categorize mother’s education as no education and with some education.

### Econometric methods

We first adopt a bivariate (nonparametric) analytical approach to understand the relationship between mother’s knowledge versus mother’s education on the distribution of HAZ and WHZ scores using kernel density plots. We also report descriptive statistics that show the means comparisons of variables by maternal education and maternal nutrition-related knowledge.

#### Econometric model specification

The analytical model employed for this study is a production function similar to the one applied in Rosenzweig and Schultz [48]. The child nutrition outcomes *N* of child *i* in household *j* depend on mother’s nutrition knowledge K, a set of maternal inputs *Y*, observable individual child’s characteristics *I*, household characteristics *H*, and regional characteristics *G*. This mathematical relationship is specified as:

$$N_{ij}=f\left(K_{ij},Y_{ij},I_{ij},H_{ij},G_{ij}\right)+\varepsilon_{ij}$$ (1)

where *N*_*ij*_ is the child nutrition outcomes measured by HAZ and WHZ as indicators for stunting and wasting, respectively. K_*ij*_ is mother’s nutrition knowledge index vector. *Y*_ij_ includes mother’s education, mother’s age at first birth, and the number of children she has borne. Vector *I*_*i*j_ includes child’s sex, child’s age and whether child is from multiple birth. The household characteristics vector *H*_*ij*_ includes household size and wealth status; while *G*_*ij*_ captures the geographic location (zone) where a child grows up (six dummy variables for zones). *Ɛ*_*ij*_ is a vector representing the net effect of all other relevant unobserved factors.

This relationship is expressed by the linear function:

$$N=\alpha_{1}+\gamma_{1}K+\beta_{1}Y+\beta_{2}I+\beta_{3}H+\beta_{4}G+\varepsilon_{1}$$ (2)

Where *N* is child nutrition outcomes and mother’s nutrition knowledge *K*. *Y*, I, H and G represent other determinants as specified in Eq (1)). To test whether the association of mother’s knowledge with HAZ and WHZ is different at the different levels of mother’s education, we add interaction terms of knowledge and different levels of mother’s education E. In particular, we estimate the following specification:

$$N=\alpha_{1}+\beta_{1}K+\beta_{2}K*E+\beta_{3}Y+\beta_{4}I+\beta_{5}H+\beta_{6}G+\varepsilon_{1}$$ (3)

Mother’s nutrition-related knowledge K is an index constructed based on key nutrition and health information described above. The levels of mother’s education used in the empirical model are no education, primary education, secondary education, and tertiary education.

## Results

This section presents the results: first, of the descriptive statistics of key variables used in analysis; second, of the bivariate association between child’s nutritional outcomes, mother’s knowledge and mother’s education; and third, of the determinants of child nutrition outcomes as measured by HAZ and WHZ.

### Descriptive results

Results in Table 1 show that mothers with knowledge of important of colostrum to new born are 38% and those who continued breastfeeding their babies after 6-month-old are about 76%. Information on diarrhea prevention and treatment using ORS was received by about 77% of the mothers, while only 10% of them had received information about the importance of family planning. About 42% have knowledge of child immunization.

**Table 1:** Descriptions of variables used for mother’s nutrition-related knowledge index.

Knowledge of	Definition and measurement	All (%)	North (%)	South (%)	No Education (%)	with Education (%)
Importance of colostrum	Mothers who gave only breastmilk (colostrum) in the first 3 days of child’s life.	37.91	34.20	49.10	27.87	50.48
Continued breastfeeding	Mothers who continued breastfeeding their children 6 months and above.	76.91	82.09	61.24	83.66	68.45
Diarrhea prevention and treatment using ORS	Mothers who received information about prevention and treatment of diarrhea using ORS.	77.07	78.18	73.70	75.87	78.57
Family planning	Mothers who received family planning information and were correct when asked about their ovulation period.	10.20	4.69	26.87	2.62	19.70
Immunization	Mothers whose children received at least 3 types of vaccination	42.44	31.21	76.38	20.12	70.41

**Source:** Authors’ computation from the Nigerian Demographic and Health Survey (NDHS), 2013. Percentage (%)

We also disaggregate mother’s nutrition-related knowledge variables by region and by education level. We find that mothers in the south have higher knowledge in all nutrition-related knowledge variables such as importance of colostrum, continued breastfeeding, diarrhea prevention and treatment using ORS, family planning and immunization. Similarly, mothers with some education have higher knowledge of importance of colostrum, and family planning and immunization than those mothers with no education.

The summary statistics results in Table 2 show the mean values for HAZ (-1.32) and WHZ (-0.94), suggesting that more than one out of three children are at least at risk of being stunted and close to one out of three is at risk of being wasted. Stunting prevalence among children aged 6–23 months in our sample is about 37%, while wasting prevalence among them is substantially high (24%). An average woman in rural Nigeria starts to give birth at age 18. This may account for why the mean for women years of education is low (3.71), suggesting that rural women in Nigeria, on average, do not have up to four years level of education. The result further shows that more than half of the mothers have never attended school, and only about 20% completed primary education.

**Table 2:** Descriptive statistics by mother’s knowledge and education.

Variable	Mother’s Knowledge		Difference test	Mother’s Education		Difference test	Pool
	Low	High		Low	High		
**Outcome Variables**							
Height-for-age z-scores (index)	-1.73	-0.87	-0.86***	-1.70	-0.69	-1.01***	-1.32
	(0.040)	(0.040)		(0.038)	(0.044)		(0.029)
Weight-for-height z-scores (index)	-1.03	-0.84	-0.19***	-1.03	-0.79	-0.23***	-0.94
	(0.032)	(0.029)		(0.030)	(0.033)		(0.022)
Prevalence of Stunting (HAZ <-2SD) (%)	46	27		45	22		37
Prevalence of Wasting (WHZ <-2SD) (%)	28	19		27	18		24
**Mother characteristics**							
Mother’s knowledge (index)	-1.09	1.09	-2.09***	-0.49	0.80	-1.28***	0.00
	(0.008)	(0.018)		(0.018)	(0.028)		(0.018)
Mother’s education (years)	1.45	6.19	-4.74***	0.37	9.19	-8.82***	3.72
	(0.06)	(0.102)		(0.021)	(0.068)		(0.070)
Mother had no education (0/1)	0.79	0.31	0.48***	0.90		0.90***	0.56
Mother had primary education (0/1)	0.14	0.26	-0.12***	0.10	0.36	-0.25***	0.20
Mother had secondary & above education (0/1)	0.07	0.43	-0.36***		0.64	-0.64***	0.24
Mother’s age (years)	27.72	28.37	-0.65***	28.09	27.92	0.18	28.03
	(0.142)	(0.139)		(0.131)	(0.154)		(0.099)
Mother’s age at first birth (years)	17.95	19.48	-1.53***	17.87	20.01	-2.14***	18.68
	(0.065)	(0.085)		(0.061)	(0.096)		(0.054)
Number of children (numbers)	4.38	3.86	0.51***	4.55	3.45	1.10***	4.13
	(0.053)	(0.051)		(0.049)	(0.053)		(0.038)
**Child characteristics**							
Child is a boy (0/1)	0.49	0.53	0.51***	0.50	0.53	-0.01**	0.51
Child’s age (months)	13.20	14.37	-1.17***	13.70	13.85	-0.15	13.76
	(0.093)	(0.106)		(0.088)	(0.119)		(0.071)
Child from multiple birth (0/1)	0.01	0.03	-0.02***	0.02	0.02	-0.01	0.02
Child’s birthweight is > 2.49 kg (0/1)	0.80	0.87	-0.07***	0.81	0.88	-0.08***	0.84
**Household and region characteristics**							
Household members (numbers)	7.34	6.76	0.58***	7.48	6.38	1.10***	7.07
	(0.067)	(0.069)		(0.064)	(0.072)		(0.049)
Poorest tercile wealth index (0/1)	0.81	0.37	0.42***	0.80	0.28	0.52***	0.61
Middle tercile wealth index (0/1)	0.14	0.30	-0.17***	0.15	0.32	-0.17***	0.22
Wealthiest tercile wealth index (0/1)	0.06	0.31	-0.25***	0.05	0.40	-0.35***	0.18
North Central (0/1)	0.11	0.21	-0.09***	0.12	0.22	-0.11***	0.16
North East (0/1)	0.25	0.21	0.04***	0.29	0.14	0.14***	0.23
North West (0/1)	0.54	0.16	0.38***	0.52	0.11	0.41***	0.36
South East (0/1)	0.01	0.10	-0.09***	0.01	0.13	-0.12***	0.05
South South (0/1)	0.05	0.23	-0.18***	0.04	0.28	-0.24***	0.13
South West (0/1)	0.04	0.09	-0.05***	0.03	0.11	-0.08***	0.06
**Number of observations**	**2,583**	**2,358**		**3,068**	**1,873**		**4,941**

**Source:** Authors’ computation from the Nigerian Demographic and Health Survey (NDHS), 2013

**Note:** The significance tests between recipients and non-recipients are the t-test for continuous variables and the

Pearson chi2 test for categorical variables. Standard deviations are presented in parentheses.

*** p < 0.01.

**p < 0.05.

* p < 0.10.

### Bivariate results

**Fig 1:**
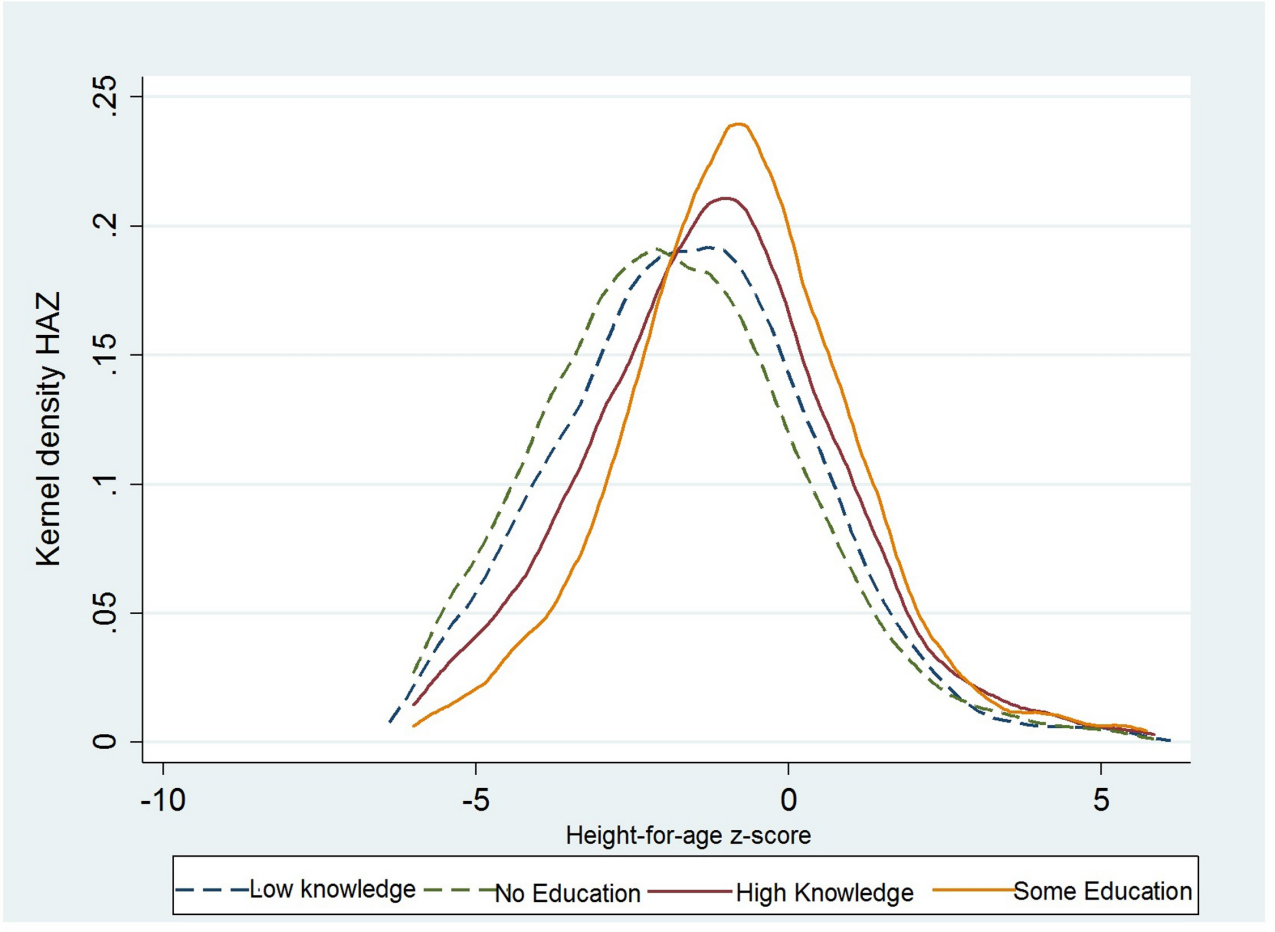
Kernel density plots of the distribution of child’s HAZ scores by mother’s knowledge and mother’s education.

**Fig 2:**
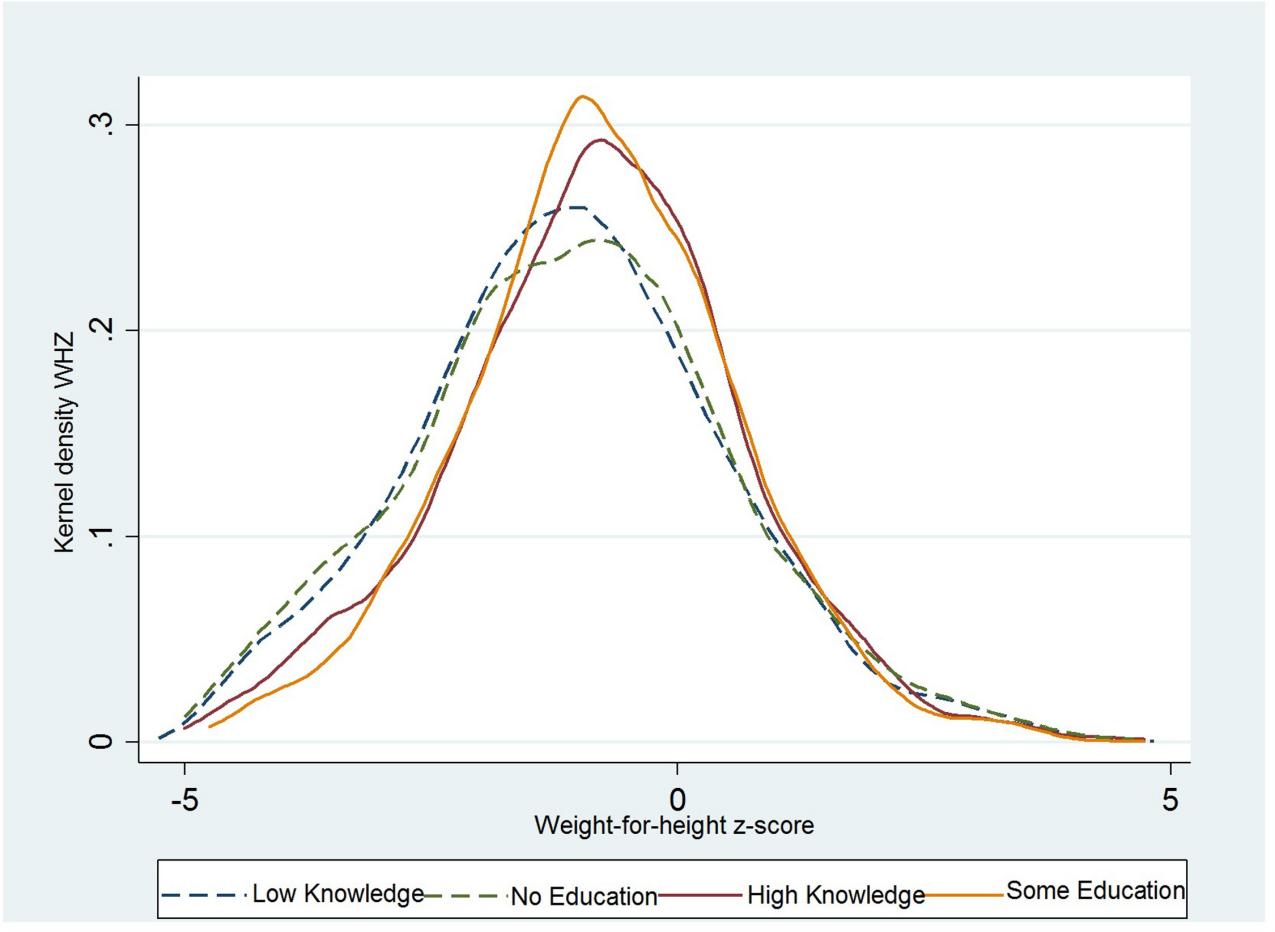
Kernel density plots of the distribution of child’s WHZ scores by mother’s knowledge and mother’s education.

The kernel density plots of the distribution of HAZ (Fig 1) and WHZ (Fig 2) for children aged 6–23 months are presented below. The results show that children’s HAZ and WHZ distributions both shift to the right for mothers with high knowledge and mothers with some education. Hence, a positive association exists between HAZ, WHZ, and mother’s knowledge and mother’s education. Moreover, this difference can be detected not only on average but also across the entire distribution of child HAZ and WHZ.

### Regression results of role of mother’s knowledge on Child HAZ and WHZ

The OLS estimates of Eqs (2) and (3) are reported in Tables 3 and 4 for the determinants of young child HAZ and WHZ, respectively. Both Tables present the estimated association of mother’s nutrition knowledge, maternal and child characteristics, household and regional characteristics with child nutritional outcomes. Five models are estimated. First, we estimate the association of mother’s nutrition knowledge with child HAZ and WHZ as a base model. The second model is mother’s knowledge-education model which controls both mother’s nutrition knowledge and education variables. The third model estimates the association of mother’s knowledge after controlling for maternal, child, household and regional characteristics. To better understand at what level of education is the association of mother’s education most significant with child nutrition outcomes, we estimate a model with mother’s level of education. To test whether the association of mother’s knowledge on HAZ and WHZ is different at the different levels of mother’s education, we estimate a model with interaction association of mother’s knowledge and levels of mother’s education.

**Table 3:** Regression results of the role of mother’s knowledge on HAZ in young children.

Variables	(1)	(2)	(3)	(4)	(5)
Mother’s knowledge (index)	0.337***	0.147***	0.141***	0.142***	0.191***
	(0.022)	(0.026)	(0.027)	(0.027)	(0.042)
Mother’s education (years)		0.091***	0.016**		
		(0.007)	(0.008)		
**Mother had no education (Base)**					
Mother had primary education				0.045	0.032
				(0.078)	(0.079)
Mother had secondary education				0.161*	0.164*
				(0.085)	(0.090)
Mother had tertiary education				0.304*	0.376*
				(0.179)	(0.230)
Mother’s knowledge index *X* Primary education					-0.090
					(0.064)
Mother’s knowledge index *X* Secondary education (0/1)					-0.086
					(0.059)
Mother’s knowledge index *X* Tertiary education (0/1)					-0.113
					(0.124)
Mother’s age at first birth (years)			-0.010	0.015**	0.015**
			(0.012)	(0.008)	(0.008)
Number of children (numbers)			-0.063**	-0.062**	-0.064**
			(0.027)	(0.027)	(0.027)
Child is a boy (0/1)			-0.371***	-0.371***	-0.372***
			(0.053)	(0.053)	(0.053)
Child’s age (months)			-0.115***	-0.115***	-0.115***
			(0.006)	(0.006)	(0.006)
Child’s age squared (months)			0.003*	0.002*	0.002*
			0.001	(0.001)	(0.001)
Child from multiple birth (0/1)			-0.614***	-0.617***	-0.638***
			(0.211)	(0.211)	(0.213)
Child’s birthweight > 2.49 kg (0/1)			0.317***	0.319***	0.313***
			(0.073)	(0.073)	(0.073)
**Poorest tercile wealth index base**					
Middle tercile wealth index (0/1)			0.038	0.043	0.036
			(0.075)	(0.075)	(0.075)
Wealthiest tercile wealth index (0/1)			0.223**	0.226***	0.228***
			(0.087)	(0.086)	(0.086)
**South West (Base)**					
North Central (0/1)			0.013	0.010	-0.002
			(0.117)	(0.117)	(0.117)
North East (0/1)			-0.456***	-0.464***	-0.470***
			(0.120)	(0.120)	(0.120)
North West (0/1)			-1.154***	-1.164***	-1.165***
			(0.121)	(0.122)	(0.122)
South East (0/1)			0.197	0.192	0.194
			(0.140)	(0.140)	(0.140)
South South (0/1)			0.156	0.153	0.152
			(0.121)	(0.121)	(0.121)
Constant	-1.315***	-1.660***	0.192	0.211	0.245
	(0.029)	(0.040)	(0.218)	(0.221)	(0.222)
R-squared	0.042	0.074	0.209	0.209	0.210
Number of observations	4,941	4,941	4,941	4,941	4,941

**Source:** Authors’ computation from the Nigerian Demographic and Health Survey (NDHS), 2013

**Note:** HAZ = Height-for-age z-score; Robust standard errors in parentheses.

*** p<0.01,

** p<0.05,

* p<0.10.

**Table 4:** Regression results of the role of mother’s knowledge on WHZ in young children.

Variables	(1)	(2)	(3)	(4)	(5)
Mother’s knowledge (index)	0.115***	0.086***	0.060***	0.145***	0.083**
	(0.017)	(0.021)	(0.022)	(0.028)	(0.035)
Mother’s education (years)		0.014***	0.008		
		(0.005)	(0.007)		
**Mother had no education (Base)**					
Mother had primary education				0.045	0.107
				(0.078)	(0.079)
Mother had secondary education				0.160*	0.049
				(0.085)	(0.194)
Mother had tertiary education				0.325*	0.162**
				(0.179)	(0.066)
Mother’s knowledge index *X* Primary education					-0.045
					(0.051)
Mother’s knowledge index *X* Secondary education (0/1)					-0.054
					(0.050)
Mother’s knowledge index *X* Tertiary education (0/1)					0.035
					(0.092)
Mother’s age at first birth (years)			0.004	0.029***	-0.006
			(0.006)	(0.010)	(0.008)
Number of children (numbers)			0.014	-0.063**	0.013
			(0.022)	(0.027)	(0.022)
Child is a boy (0/1)			-0.109**	-0.378***	-0.116***
			(0.044)	(0.053)	(0.044)
Child’s age (months)			0.020***	-0.115***	0.021***
			(0.005)	(0.006)	(0.005)
Child’s age squared (months)			0.003***	0.015*	0.005
			(0.001)	(0.008)	(0.006)
Child from multiple birth (0/1)			-0.615***	-0.605***	-0.656***
			(0.176)	(0.220)	(0.176)
Child’s birthweight > 2.49 kg (0/1)			0.281***	0.316***	0.271***
			(0.062)	(0.073)	(0.062)
**Poorest tercile wealth index base**					
Middle tercile wealth index (0/1)			-0.012	0.038	-0.024
			(0.060)	(0.075)	(0.060)
Wealthiest tercile wealth index (0/1)			0.031	0.229***	0.037
			(0.074)	(0.086)	(0.073)
**South West (Base)**					
North Central (0/1)			0.030	0.009	0.017
			(0.088)	(0.117)	(0.086)
North East (0/1)			-0.125	-0.457***	-0.115
			(0.091)	(0.120)	(0.091)
North West (0/1)			-0.017	-1.162***	0.003
			(0.093)	(0.122)	(0.093)
South East (0/1)			-0.104	0.191	-0.101
			(0.107)	(0.140)	(0.107)
South South (0/1)			0.066	0.155	0.051
			(0.091)	(0.121)	(0.092)
Constant	-0.939***	-0.992***	-1.325***	0.225	-1.331***
	(0.022)	(0.031)	(0.178)	(0.221)	(0.182)
R-squared	0.009	0.010	0.026	0.210	0.028
Number of observations	4,941	4,941	4,941	4,941	4,941

**Source:** Authors’ computation from the Nigerian Demographic and Health Survey (NDHS), 2013

**Note:** WHZ = Weight-for-height z-score; Robust standard errors in parentheses.

*** p<0.01,

** p<0.05,

* p<0.10.

The first column in Tables 3 and 4 presents results based on the base model. The results show that mother’s nutrition-related knowledge has a significant and positive association with child HAZ and WHZ. Also, a one-point increase in the nutrition knowledge index is associated with 0.34 SD and 0.12 SD increase in child HAZ and WHZ scores, respectively. Although the significance and positive association with mother’s nutrition knowledge in the model does not change when we include mother’s education into the model (see second column in Tables 3 and 4), it reduces the magnitude of the nutrition knowledge index for both child HAZ and WHZ. We further control maternal, child, household and regional characteristics in model three; the results on the association of mother’s nutrition knowledge are virtually the same whereas the effect of mother’s education on child WHZ become statistically insignificant when we control for these characteristics. The fourth and fifth column in Tables 3 and 4 presents results for different levels of mother’s education, and the interaction of mother’s nutrition knowledge with the level of mother’s education. Primary school education does not seem to have a significant association with child nutritional outcomes while mother’s education level beyond primary school has a positive and statistically significant association with child HAZ and WHZ (see fourth column in Tables 3 and 4). On average, children from mothers who completed secondary and tertiary education are associated with 0.16 and 0.30 points higher HAZ than those of children from mothers with no education. Similarly, children from these mothers are associated with 0.16 and 0.33 points higher WHZ than those from mothers with no education. The coefficient of the interaction of education level (dummy) and knowledge shows no significant association with child HAZ and WHZ scores (see fifth column in Tables 3 and 4).

When we look at the association of number of children, the results show that number of children in the household has a statistically significant association with child HAZ. Turning to the association of child characteristics with child nutritional outcomes, consistent with [43] the results show that boys are systematically more undernourished than girls. Their HAZ and WHZ scores are, on average, 0.37 and 0.12 points lower than those of girls, respectively. A one-child increase in household above the average tend to decrease a child’s HAZ scores by 0.03 SD. The results show that child HAZ scores deteriorate up to the age of 14 months and slightly improve afterwards. We also show that children from multiple birth has a negative and significant association with child HAZ and WHZ. Children from multiple birth are associated with 0.64 z-scores shorter and 0.66 z-scores thinner than children from a single birth. Similarly, our results show that children with average birthweight are less undernourished than children with low birthweight.

To capture regional difference in child nutritional outcomes, we also included regional dummies. There are significance variations in child nutritional outcomes across regions. The negative and statistically significant coefficients for region dummy for north-east and north-west regions suggest that children in these regions have significantly lower nutritional outcomes compared with children in south-west. However, there was no significant difference in child nutritional outcomes across north-central and south-east as compared to south-west.

## Discussion

Stunting prevalence among the sample is about the national average, however, wasting prevalence is almost double the national average for under-fives. From the magnitude of the association of mother’s knowledge with child HAZ and WHZ in our analysis, it is evident that mother’s knowledge of nutrition and health is very helpful in safeguarding young children from occasions that reduce children HAZ and WHZ scores. Occasions such as diarrhea episodes in children as a result of poor hygiene and early introduction of liquid substances other than breastmilk to young children, poor complementary infant feeding, and more under-fives per mother due to limited knowledge of family planning methods all produce stunting and wasting in young children. These results are consistent with some studies in developing countries. For example, Webb and Block [49, 50] found a significant association between maternal nutrition knowledge and wasting in Indonesia but not stunting. Likewise, Appoh and Krekling [42] in Ghana found associations between mother’s nutrition knowledge and underweight, which measures both stunting and wasting. Mothers with above primary level education can significantly increase HAZ scores, (stunting reduction) in children and WHZ scores (wasting reduction) in children.

Studies [17, 31] show that in a setting where illiteracy rate is higher, low level of parental education, typically below junior secondary, has no significant effect on child undernutrition. Given the present level of mother’s formal education in the studied population, further gains are unlikely to reinforce mother’s nutrition knowledge for the purposes of reducing undernutrition.

Furthermore, having more children is positively associated with stunting rate in children. More children, particularly under five children, per mother tend to increase intra-household competition for childcare resources such that a child is denied adequate nutritional care [51]. Also, older children tend to get less care as the result further suggests; older children have lower HAZ scores than younger ones. This present study and some other studies suggest that delaying age at first birth by mother is positively association with better child nutritional outcomes. A positive association has been shown to exist between children from multiple birth and childhood undernutrition. For example, a study in south-west Nigeria has shown that infants from multiple birth are associated with adverse perinatal outcomes, especially in resource-poor environments [52]. In some African countries, including Nigeria as this study reveals, young boys are found to suffer from malnutrition than young girls [53, 54]. Our results also show that children with average birthweight and children from a single birth are less malnourished than children with low birthweight and those from multiple birth.

## Conclusions

The determinants of child nutrition outcomes explored in this study have great policy implications for Nigeria and are also applicable to other countries with similar characteristics. The findings emanating from this study improve our understanding of the independent role that mother’s nutrition-related knowledge can play on child nutrition outcomes. Mother’s knowledge of food choices, feeding, and health care seeking are vital for producing good nutrition outcomes for young children. The present level of mother’s educational attainment in rural Nigeria is not sufficient to reinforce knowledge in producing better nutrition outcomes for children. In such populations with limited access to formal education, mother’s knowledge of health and nutrition may substitute for education in reducing undernutrition in young children. Particular effort should therefore be made to ensure this knowledge is rightly acquired. Other means of knowledge acquisition for mothers who do not have a formal education or cannot complete education above primary school should be employed. More importantly, programs should identify which child care practices should be encouraged to enhance maternal and child nutrition and health; promoting such could mitigate the negative association of mother’s lack of education with children’s health. Finally, adult education through behavioral change communications would be an effective means for reaching out to poor and less educated Nigerian women.

## Supporting information

S1 File Mother’s knowledge index construction using principal component analysis. (DOCX)
