# Correction: Mother’s nutrition-related knowledge and child nutrition outcomes: Empirical evidence from Nigeria

**DOI:** 10.1371/journal.pone.0215110

**Published:** 2019-04-04

**Authors:** Olusegun Fadare, Mulubrhan Amare, George Mavrotas, Dare Akerele, Adebayo Ogunniyi

There are errors in the Author Contributions. The correct contributions are: Conceptualization: OF MA GM DA. Data curation: OF MA DA AO. Formal analysis: OF MA GM. Investigation: OF MA GM. Methodology: OF MA. Software: OF MA. Supervision: MA GM. Writing—original draft: OF MA GM DA AO. Writing—review & editing: OF MA GM DA.

There are errors in the Funding statement. The correct Funding statement is as follows: The authors received no specific funding for this work.

S1 File appears incorrectly. Please view the correct S1 File below.

## Supporting information

S1 File Mother’s knowledge index construction using principal component analysis. (DOCX)
