# Supplementary material for: Mother’s nutrition-related knowledge and child nutrition outcomes: Empirical evidence from Nigeria
Source: PLoS One. 2019 Feb 28;14(2):e0212775. doi: 10.1371/journal.pone.0212775 (PMC6394922; doi:10.1371/journal.pone.0212775)
Supplement: S1 File — (DOCX) [file pone.0212775.s001.docx]

**https://www.thebalancecareers.com/thmb/UXAR16ug3zB8m2xyFqR9y_773Ns=/1000x0/filters:no_upscale():max_bytes(150000):strip_** **
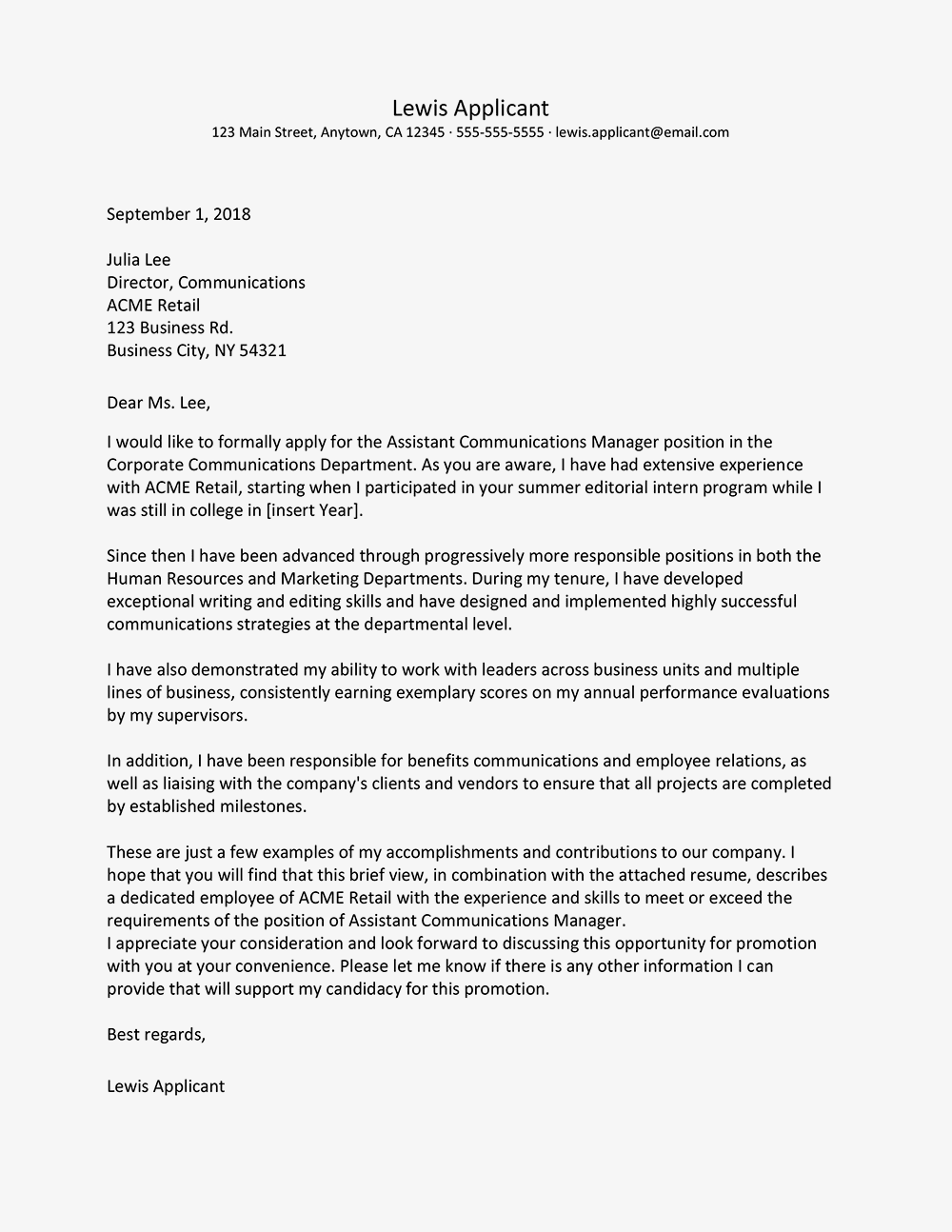
 icc()/2061682v1-5ba55b1446e0fb0025cdd37b.png**
