# Supplementary material for: Correction: Mother’s nutrition-related knowledge and child nutrition outcomes: Empirical evidence from Nigeria
Source: PLoS One. 2019 Apr 4;14(4):e0215110. doi: 10.1371/journal.pone.0215110 (PMC6448814; doi:10.1371/journal.pone.0215110)
Supplement: S1 File — (DOCX) [file pone.0215110.s001.docx]

**S1 File. Mother’s Knowledge Index construction using Principal Component Analysis**

Given a data matrix with variables (importance of colostrum, continued breastfeeding, Diarrhea prevention and treatment using ORS, family plaining and immunization) and 4,941 observations, we can write it as the following:

$Y_{1}= e_{11}X_{1}+ e_{21}X_{2}+\ldots+ e_{p1}X_{p}$;

$Y_{2}= e_{12}X_{1}+ e_{22}X_{2}+\ldots+ e_{p2}X_{p}$ ;

$Y_{p}= e_{1p}X_{1}+ e_{2p}X_{2}+\ldots+ e_{pp}X_{p}$ where *i* = 1-n observations, *j* = 1-p

Symmetrically, the objective of the PCA is to scheme the data matrix *X* from *p* dimensions (i.e., the five variables for mother’s knowledge) to a smaller dimension *k*, Mother’s Nutrition-related Knowledge Index, where k << *p* (1 index <<4 indicators), meanwhile keeping as much information (i.e., variance maximization) as possible in this dimension-reduced data matrix with the size *n* by *k*4. Specifically, the PCA method replaces an outsized number of correlated variables ($X_{1}$, …,$X_{p}$) with a smaller number of uncorrelated variables (*PC*1, …, *PC*k). Mathematically, the first principal component is a linear combination of $X_{1}$ to $X_{p}$ observed variables that accounts for the largest variance among them:

$$PC1 = a_{1}X_{1} + a_{2}X_{2}+ \ldots+ a_{p}X_{p}$$

Where the vector of coefficient *a* (j = 1…p) is termed loading vector and is normalized to avoid inflating the variance of *PC*1. By the same token, the second principal component (*PC*2) is another linear combination of X variables that gives explanations for the largest variance among them, however, with a constraint; *PC*2 is required to be orthogonal to *PC*1. Theoretically, we are able to track as many principal components as the number of variables in the data matrix X. But in practice, we search for a much smaller number of principal components (PCs) that is able to capture as much as information from the original set of *X* variables.

**Principal components (eigenvectors)**

| **Variable** | **Component** | | | | |  |
| --- | --- | --- | --- | --- | --- | --- |
|  | **1** | **2** | **3** | **4** | **5** | **Kaiser-Meyer-Olkin measure** |
| Importance of colostrum | 0.523 | -0.380 | 0.318 | 0.181 | 0.670 | 0.549 |
| Continued breastfeeding | -0.496 | 0.447 | -0.032 | 0.541 | 0.510 | 0.557 |
| Diarrhea prevention and  treatment using ORS | 0.387 | 0.540 | -0.478 | -0.453 | 0.353 | 0.554 |
| Family planning | 0.523 | 0.140 | -0.330 | 0.684 | -0.358 | 0.574 |
| Immunization | 0.235 | 0.587 | 0.749 | -0.042 | -0.195 | 0.564 |
| Overall |  |  |  |  |  | 0.559 |

| **Variables** | **Eigenvalue** | **Proportion** |
| --- | --- | --- |
| Component 1 | 1.319 | 0.264 |
| Component 2 | 1.034 | 0.208 |
| Component 3 | 0.957 | 0.192 |
| Component 4 | 0.872 | 0.175 |
| Component 5 | 0.8112 | 0.162 |
